# Plocabulin, a novel tubulin-binding agent, inhibits angiogenesis by modulation of microtubule dynamics in endothelial cells

**DOI:** 10.1186/s12885-018-4086-2

**Published:** 2018-02-07

**Authors:** Carlos M. Galmarini, Maud Martin, Benjamin Pierre Bouchet, María José Guillen-Navarro, Marta Martínez-Diez, Juan Fernando Martinez-Leal, Anna Akhmanova, Pablo Aviles

**Affiliations:** 1 R&D Area, PharmaMar S.A, Avda. de los Reyes 1, 28770 Colmenar Viejo, Madrid, Spain; 2 Cell Biology, Faculty of Science, Utrecht University, Padualaan 8, 3584 CH Utrecht, The Netherlands

**Keywords:** Plocabulin (PM060184), Angiogenesis inhibitors, Tubulin inhibitors, Endothelial cells, Vascular-disrupting agents, Cancer treatment

## Abstract

**Background:**

Vascular supply of tumors is one of the main targets for cancer therapy. Here, we investigated if plocabulin (PM060184), a novel marine-derived microtubule-binding agent, presents antiangiogenic and vascular-disrupting activities.

**Methods:**

The effects of plocabulin on microtubule network and dynamics were studied on HUVEC endothelial cells. We have also studied its effects on capillary tube structures formation or destabilization in three-dimensional collagen matrices. In vivo experiments were performed on different tumor cell lines.

**Results:**

In vitro studies show that, at picomolar concentrations, plocabulin inhibits microtubule dynamics in endothelial cells. This subsequently disturbs the microtubule network inducing changes in endothelial cell morphology and causing the collapse of angiogenic vessels, or the suppression of the angiogenic process by inhibiting the migration and invasion abilities of endothelial cells. This rapid collapse of the endothelial tubular network in vitro occurs in a concentration-dependent manner and is observed at concentrations lower than that affecting cell survival. The in vitro findings were confirmed in tumor xenografts where plocabulin treatment induced a large reduction in vascular volume and induction of extensive necrosis in tumors, consistent with antivascular effects.

**Conclusions:**

Altogether, these data suggest that an antivascular mechanism is contributing to the antitumor activities of plocabulin.

**Electronic supplementary material:**

The online version of this article (10.1186/s12885-018-4086-2) contains supplementary material, which is available to authorized users.

## Background

Angiogenesis is one of the critical steps required for solid tumors to grow beyond their dormant state [1–3]. During this process, endothelial cells have to disrupt the surrounding basement membrane, migrate, invade towards a pro-angiogenic stimulus, proliferate to provide additional cells that form new vessels and re-organize to form the necessary three-dimensional vessel structure [4–6]. Both, the actin and microtubule cytoskeletons play a key role in these processes as they regulate the maintenance of endothelial cell shape changes as well as endothelial cell proliferation [7, 8]. Tumor vasculature is not a simple supply line of nutrients to tumors [1, 9, 10]. It governs pathophysiology of solid tumors and thus tumor growth, invasion, metastasis and response to various therapies [11]. Consequently, it is currently accepted that inhibition of angiogenesis is an effective strategy to treat human cancers [12].

Recent studies have shown that most of the microtubule-targeting agents (MTA) present antiangiogenic and vascular-disrupting effects [13–17]. By affecting the microtubule network, MTAs inhibit endothelial cell proliferation, migration, and tube formation, and cause prominent changes in endothelial cell morphology, an action associated with rapid vascular collapse in vivo [14, 18–20]. Thus, current data suggest that MTAs would be a particularly useful class of antiangiogenic drugs as they have multiple direct actions on endothelial cells [21, 22]. Taxanes, colchicine, combretastatins and vinca alkaloids were among the first MTAs reported to have antiangiogenic or vascular-disrupting properties [17, 18, 20, 23, 24]. These observations have prompted the development of new microtubule-binding drugs with antiangiogenic activity.

Plocabulin (PM060184) is a new marine-derived drug that binds to a new site in β-tubulin, inhibiting tubulin polymerization [25–27]. The compound is currently being evaluated in Phase I/II studies in patients with advanced malignancies. The present study describes the antiangiogenic and vascular-disrupting properties of plocabulin. We show that, at picomolar concentrations, plocabulin inhibits microtubule dynamics in endothelial cells leading to alterations of cytoskeletal organisation, and thus morphology changes as well as suppression of their migration and invasion abilities. This results in tumor vascular endothelial architectural destabilization and tumor vascular collapse. The in vitro findings were confirmed in tumor xenografts where, even at doses below its maximum-tolerated dose (MTD), plocabulin treatment induced a large reduction in vascular volume and induction of extensive necrosis, consistent with antivascular effects. Altogether, these data suggest that an antivascular mechanism might also contribute to the antitumor activities of plocabulin [26, 27].

## Methods

### Reagents

High concentration rat tail Collagen I was obtained from BD Biosciences (San José, CA, USA). Phorbol 12-myristate 13-acetate (PMA), Hoechst 33,342, 3-(4,5-dimethylthiazol-2-yl)-2,5-diphenyltetrazolium bromide (MTT), mouse monoclonal anti-α-tubulin (T5168), sulforhodamine B (SRB), and phalloidin-FITC (P5282) were obtained from Sigma (St Louis, MO, USA). Human recombinant Fibroblast Growth Factor-basic (FGF) was from Peprotech (Rocky Hill, NJ, USA). Alexa 594-conjugated goat anti-mouse IgG secondary antibodies (A11032) were obtained from Molecular Probes (Rockford, IL, USA). For in vitro experiments, plocabulin (PharmaMar, Madrid, Spain) was prepared as a 1 mg/ml stock solution in DMSO and stored at − 80 °C. For in vivo experiments, lyophilized vials (1.6 mg/vial) of plocabulin (PharmaMar) were used. For xenograft studies, potential antiangiogenic effect induced by the treatment was studied by Angiosense TM 680EX Fluorescent Imaging Agent (Perkin Elmer Inc., MA, USA).

### Cell lines and cell culture

Human Umbilical Vein Endothelial Cells (HUVECs) were obtained from Lonza (Basel, Switzerland) and grown in endothelial basal medium (EGM-2) supplemented with growth supplements: 2% Fetal Bovine Serum (FBS), human Epidermal Growth Factor (hEGF), Vascular Endothelial Growth Factor (VEGF), R3-Insulin-like Growth Factor-1 (R3-IGF-1), ascorbic acid, hydrocortisone human Fibroblast Growth Factor-Beta (hFGF-β), heparin, gentamicin/amphotericin-B (GA). Human microvascular endothelial cells (HMEC-1) were obtained from ATCC (Manassas, Virginia, USA) (CRL-3243) and grown in MCDB131 (without L-Glutamine) medium supplemented with hEGF (10 ng/ml), hydrocortisone (1 μg/ml), glutamine (10 mM) and 10% FBS. Only low passage cells (between passages 3 and 7) were used. For microtubule dynamics, EB3-GFP plasmid was nucleofected using Amaxa technologies with the HUVEC nucleofector kit (Lonza) according to the manufacturers’ protocols. For xenograft experiments, we used 2 human derived cell lines: NCI-H460 non-small cell lung carcinoma (HTB-177™) and MDA-MB-231 breast adenocarcinoma (CRM-HTB-26™), both from ATCC (Manassas). Before animal inoculation, cells were maintained in vitro at 37 °C with 5% CO2 in Dulbecco’s Modified Eagle’s Medium (Sigma-Aldrich) and passaged every 3 to 5 days upon reaching confluence.

### Detection of microtubule and actin cytoskeletons by immunofluorescence staining

Endothelial cells were treated with plocabulin at different concentrations for 6 h, 24 h and 48 h, fixed with methanol for 10 min at − 20 °C and incubated with a blocking solution (5% bovine serum albumin in PBS) for 30 min. Cells were then incubated with primary mouse anti-human α-tubulin antibody for 1 h at room temperature. After three washes with a PBS/BSA1% solution, cells were incubated with Alexa 594-conjugated goat anti-mouse IgG secondary antibody at room temperature for 1 h. Cells were then incubated with phalloidin-FITC for 1 h at 37 °C in a humid chamber. Cells were finally counterstained with addition of Hoechst 33,342 (1 μg/ml) for 5 min and mounted with Mowiol mounting medium. Pictures were taken with a Leica DM IRM fluorescence microscope equipped with a 100× oil immersion objective and a DFC 340 FX digital camera (Leica, Wetzlar, Germany).

### Profiling of angiogenesis-related proteins in cell culture supernates

The relative expression profile of 55 human angiogenesis-related proteins was performed using Proteome Profiler Human Angiogenesis Array kit (R&D Systems, NE, Minneapolis, U.S.A) after treatment of HUVEC endothelial cells with plocabulin for 24 h.

### Measurements of microtubule dynamics

Fluorescence imaging of EB3-GFP tagged microtubules in live endothelial cells was performed by confocal fluorescence illumination on a Nikon Eclipse Ti microscope equipped with a perfect focus system (Nikon, Tokyo, Japan), a spinning disc-based confocal scanner unit (CSU-X1-A1, Yokogawa, Tokyo, Japan), an Evolve 512 EMCCD camera (Photometrics, Tucson, AZ, USA) attached to a 2.0X intermediate lens (Edmund Optics, Barrington, NJ, USA) and a motorized stage MS-2000-XYZ with Piezo Top Plate (ASI), using a stage top incubator INUBG2E-ZILCS (Tokai Hit, Fujinomiya-shi, Shizuoka-ken, Japan) for 37 °C/5% CO2 incubation and 37 °C lens heating. The microscope setup was controlled by MetaMorph 7.7.11.0. Acquisitions were performed at 0.5 s interval with a 200 milliseconds exposure during 2 min using an Apo TIRF 100× NA 1.49 oil. Cells were treated with plocabulin for one hour before imaging. Kymographs of microtubule plus end dynamics were made using the MTrackJ plugin of ImageJ software (NIH, Bethesda, MA, USA) and analyzed using the same software. Only microtubule length changes ≥ 0.3 μm between two consecutive time points were considered as growth or shortening events, while changes < 0.3 μm were considered as pause event; only the events starting and finishing within the recording were analyzed. Velocity and covered distance were calculated for each growth event and were then averaged. Catastrophe frequency was calculated by dividing the number of catastrophes (transition from growth or pause to shortening) by the sum of growth and pause durations. For each condition, at least 10 microtubules per cell, in 10 cells in three independent experiments were analyzed. Comparisons between different samples were analyzed by Student’s t test. Differences were considered significant at ***P < 0.001.

### Cell viability assay

The MTT colorimetric assay was used for quantitative measurement of cell viability, as previously described [26]. The highest plocabulin concentration in the assay was 17 nM and then, serial dilutions 1/2.5 from this initial concentration were added to the cells.

### Adhesion, migration and invasion assays

For adhesion assays, HUVEC cells (75,000 cells/well) were cultured in 96-well plates covered with fibronectin (2.5 μg/ml) and collagen 0.05 μg/ml at 4 °C for 10 min. Adhesion was then allowed at 37 °C during 30 min in the absence or presence of plocabulin. After 3 washes with PBS, remaining cells were fixed with glutaraldehyde (0.1%) and stained with sulforhodamine B. As positive and negative controls, we have used MnCl_2_ (0.5 mM), latrunculin A (3 μM) and cytocalasin D (2 μM), respectively. For migration and invasion assays, 6.5 mm-diameter transwell chambers (Sigma-Aldrich, St. Louis, MO, USA) with polycarbonate membrane (pore size 8.0 μm) were used. HUVEC cells (1.5 × 105) were seeded on the upper compartment of the transwell membrane in 100 μl of serum-free culture medium containing or not plocabulin. The lower compartment (well) was filled with 600 μl of serum-free culture medium containing or not a chemoattractant (FBS 2%). For the invasion assays, the upper compartment of the transwell was previously coated with 12 μg of matrigel basement membrane matrix to create a physical barrier between the two compartments of the chamber. After 24 h of incubation, culture medium was removed from the inside of the chamber, and the non-migrated cells on the upper side of the membrane were wiped off using a cotton swab. Migrated cells on the lower side of the membrane were fixed with a glutaraldehyde 1% solution, washed and stained with sulforhodamine B following standard techniques.

### HUVEC capillary tube structures formation or destabilization in three-dimensional collagen matrices

The tube formation or destabilization assays are based on the ability of endothelial cells to form three-dimensional capillary-like tubular structures when cultured on a basement membrane matrix. HUVEC cells (2.5 × 104 cells) were serum-starved overnight, resuspended in 100 μl of medium supplemented with FBS 2% per well and plated on 96-well plates previously coated with 50 μl of Matrigel (BD Biosciences). For tube formation assays, fresh culture medium containing FBS 2% alone or FBS 2% plus plocabulin was added after 1 or 6 h of incubation at 37 °C. For tube destabilization assays, fresh culture medium containing FBS 2% alone or FBS 2% plus plocabulin was added when capillary tube structures were well established. In both cases, after 6 h and 24 h post-treatment, cells were stained with calcein-AM to measure cell viability and analyze tube formation. Cultures were observed and photographed by phase-contrast and fluorescence microscopy.

### 3D sprouting

Endothelial cell spheroids were generated overnight by culturing endothelial cells in complete medium containing 20% methylcellulose in non-adherent 96-wells plates. Harvested spheroids were then embedded into 2 mg/ml collagen gels overlaid with complete medium supplemented with 40 ng/ml FGF, 50 ng/ml PMA and with the indicated drug concentration. Angiogenic activity was quantified by measuring the cumulative length of the sprouts that had grown out of each spheroid, their mean number and length, 24 h after embedding using ImageJ software. For each condition, at least 20 spheroids were analyzed. Comparisons between different samples were analyzed by Student’s t test. Differences were considered significant at *P < 0.05, **P < 0.01 and ***P < 0.001.

### Xenograft murine models

All animal protocols were reviewed and approved according to regional Institutional Animal Care and Use Committees. Design, randomization and monitoring of experiments (including body weights and tumor measurements) were performed using NewLab Software v2.25.06.00 (NewLab Oncology, Vandoeuvre-Lès Nancy, France). Female athymic Nude-Foxn-1 nu/nu mice (Envigo, RMS Spain S.L.) between 4 to 6 weeks of age were s.c. xenografted with NCI-H460 or MDA-MB-231 cancer cells into their flank with ca. 5 × 106 cells or 7.5 × 10,6 respectively. In the NCI-H460 experiment, when tumors reached ca. 150 mm^3^, mice were intravenously administered in three consecutive weekly doses (0.08, 8 and 16 mg/kg/day) whereas the control animals received an equal volume of vehicle with the same schedule. Caliper measurements of the tumor diameters were made three times a week and tumor volumes were calculated according to the following formula: (axb2) /2, where a and b were the longest and shortest diameters respectively. Animals were humanely sacrificed when their tumors reached 2500 mm^3^ or if significant toxicity (e.g. severe body weight reduction) was observed. Differences in tumor volumes between treated and control group were evaluated using the Mann–Whitney U-test. Statistical analyses were performed by Graph Pad Prism® v5.03 (Graph Pad Software Inc. La Jolla, CA, USA). In addition, three randomly-selected H460 tumor-bearing animals were dedicated to study blood vessel density and to characterize vascular changes related with plocabulin administration by an in vivo imaging system, IVIS^®^ Spectrum (Perkin Elmer Inc., Waltham, MA, USA). When tumors reached ca. 300 mm^3^, animals (*n* = 3/group) were treated with a single dose of either placebo, or plocabulin (at 2 or 16 mg/kg) and then, they were administered via tail vein injection with AngioSense 680 EX, which is a near-infrared labeled fluorescent macromolecule that remains localized in the vasculature for extended periods of time and enables imaging of blood vessels and angiogenesis. Fluorescent signal acquisition was performed at 24 h after the treatment and AngioSense dosing. In the MDA-MB-231 experiment, six tumor bearing mice, ca. 500 mm^3^ were randomly selected 24 h after being administered with plocabulin at 16 mg/kg or placebo. The animals were sacrified by CO_2_ asphyxiation whithin their home cages, being CO_2_ flow set to displace 20% of the cage volume per minute. Death was confirmed by physical examination. Tumors were then dissected free and processed for paraffin embedding and sectioning, serial sections were cut and stained with hematoxylin/ eosin for histology evaluation.

## Results

### Effects of plocabulin on endothelial cell morphology and microtubule and actin cytoskeletons

**Fig. 1:**
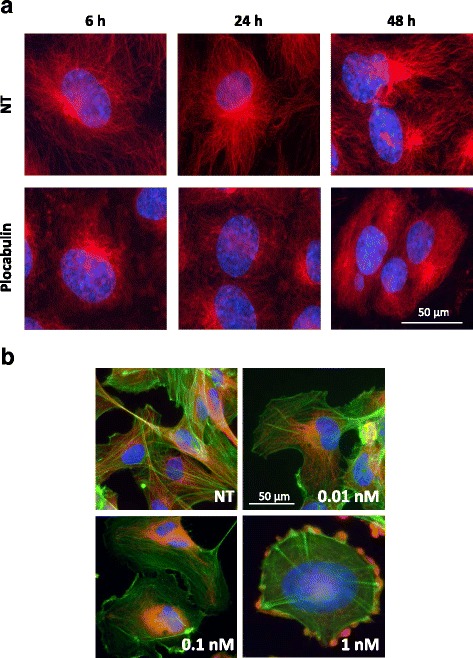
Effects of plocabulin on HUVEC cell morphology and microtubule mass by fluorescence microscopy. **a** HUVEC endothelial cells were cultured in the absence or presence of plocabulin 0.1 nM at different time intervals. Cells were then stained for α-tubulin (red) and nuclei (blue). **b** HUVEC endothelial cells were cultured in the absence or presence of increasing concentrations of plocabulin for 24 h. Representative images of each treatment conditions are shown

Tubulin cytoskeleton in untreated HUVECs cells is characterized by cytoplasmic microtubules radiating from a central point (microtubule organizing center) to the cell periphery (Fig. 1, red). Treatment of HUVEC cells with different concentrations of plocabulin resulted in depolymerization of the microtubule network (representative images are shown in Fig. 1a). At low concentrations (0.01 nM), these effects were observed after 48 h of incubation with the drug while, at higher levels (0.1–1 nM), they were observed as soon as after 6 h of treatment (Additional file 1: Figure S1A). Similar results were obtained with immortalized human HMEC-1 cells (Additional file 1: Figure S1B). Of note, in these cells, the effects of plocabulin 0.01 nM were observed even after only 6 h of drug treatment. We have also evaluated the effects on the morphology and the actin cytoskeleton of plocabulin on HUVEC cultures (Fig. 1b). Differently from untreated cells, plocabulin-treated cells showed a rounded morphology with well-developed actin filament structure similar to the “dense peripheral bands” observed in mature endothelial cells [28, 29]. Moreover, plocabulin also induced endothelial cell retraction and rounding and plasma membrane blebbing (Fig. 1b). All these effects were dependent on concentration and time of incubation: the higher the concentration, the faster they appeared. Again, these effects were observed at similar levels after treatment of HMEC-1 cells with the drug (Additional file 1: Figure S1). We have finally analyzed the expression profiles of 55 angiogenesis-related proteins in supernatants of HUVEC cultured cells in the absence or presence of plocabulin 0.05 nM for 24 h using a membrane-based sandwich immunoassay. The analyses showed that none of these proteins were altered in HUVEC cells after treatment (data not shown).

### Effects of plocabulin on microtubule dynamics of endothelial cells

**Fig. 2:**
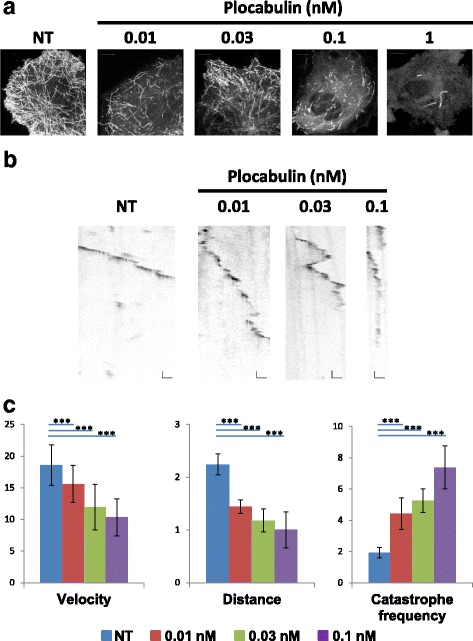
Effects of plocabulin on microtubule dynamics in HUVEC cells. **a** EB3-GFP maximum intensity projection for two minutes acquisition with two frames/s in each condition; EB3-GFP was transfected into HUVEC cells as describe in material and methods. **b** Representative kymographs of EB3 dynamics for each condition. Horizontal and vertical bars represent 1 um and 10 s respectively. **c** Histograms representing the mean velocity (μm/min) and distance of growth events (μm), as well as the mean catastrophe frequency (min^− 1^) in each condition. Data are shown as mean ± SD. Comparisons between different samples were analyzed by Student’s t test. Differences were considered significant at ***P < 0.001

To test the effect of plocabulin on microtubule dynamics in living endothelial cells, we ectopically expressed EB3-GFP using Amaxa nucleofector technology in HUVEC cells. Twenty-four hours after transfection, endothelial cells were treated with different concentrations of plocabulin for one hour. Treatment with DMSO was used as a non-treated condition. Microtubule plus-end dynamics was then live-recorded using spinning-disk confocal microscopy. As shown in Fig. 2a, with the maximum intensity projection of EB3-GFP signal during 2 min acquisition, microtubules were detected in the presence of plocabulin concentrations ranging from 0.01 nM to 0.1 nM, whereas addition of 1 nM induced an almost complete suppression of GFP-labeled microtubule plus-end signals. Plocabulin-treated cells showing remaining microtubules were then analyzed for microtubule dynamics. Kymographs were drawn for at least 100 MTs in each condition and subsequently analyzed (Fig. 2b and c and Additional file 2: Table S1). Plocabulin treatment significantly reduced the velocity and the covered distance of growth events in a concentration-dependent manner. Addition of this drug also caused a strong increase in catastrophe frequency.

### Effects of plocabulin on adhesion, migration and invasiveness of HUVEC cells

**Fig. 3:**
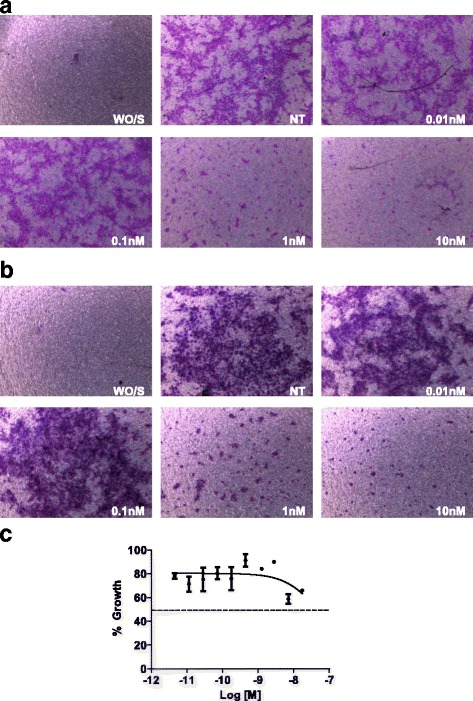
Plocabulin inhibits the migration and invasion capabilities of HUVEC cells. **a** For migration experiments, HUVEC cells were seeded in serum-free medium into the upper compartment of transwell inserts and incubated at 37 °C for 24 h in the absence (WO/S) or presence of FBS 2% (NT) or the indicated concentrations of plocabulin. After removing non-migrated cells from the upper compartment, migrated cells in the lower compartment were stained with sulforhodamine B. **b** For invasion experiments, the porous membranes of transwell inserts were pre-coated with matrigel basement membrane (12 μg); HUVEC cells were then seeded in serum-free medium (WO/S) into the upper compartment and incubated at 37 °C for 24 h in the absence (NT) or presence of the indicated concentrations of plocabulin. After removing non-invasive cells from the upper compartment, migrated cells in the lower compartment were stained with sulforhodamine B. **c** Antiproliferative activity of plocabulin in HUVEC cells after 24 of incubation. Cell viability was measured by MTT assay

HUVEC cells were left to adhere to an extracellular matrix composed of fibronectin and type I collagen for 10 min and were then exposed for a short period (30 min) to plocabulin ranging from 1 pM to 10 nM. No detachment of cells from the extracellular matrix was observed (data not shown). We then evaluated the in vitro effect of plocabulin on endothelial cell migration and invasion using transwell chambers and HUVEC primary cells. As shown in Fig. 3a, HUVEC cells showed very low basal levels of migration in serum-free medium, but migration was greatly increased in the presence of FBS 2% added to the lower chamber as chemo-attractant. HUVEC cells were then cultured in the presence of chemo-attractant and treated with different concentrations (0.01, 0.1, 1 and 10 nM) of plocabulin. As shown in Fig. 3a, the drug inhibited the migration of endothelial cells in a concentration-dependent manner. Concentrations higher than 0.1 nM resulted in a nearly complete abrogation of cell migration. For invasion experiments, the porous membrane of transwell chambers was pre-coated with a layer of Matrigel mimicking the physiological basement membrane. The results obtained were similar to those described for the migration assays (Fig. 3b). Plocabulin completely inhibited cell invasion at concentrations higher than 0.1 nM. To discard that the inhibition of cell migration and invasion exerted by plocabulin were due to a direct cytotoxic activity, cell survival of HUVEC cells was analyzed in concentration-response curves using a standard MTT method. As shown in Fig. 3c, at the effective concentrations used in the migration and invasion assays, plocabulin was not cytotoxic against HUVEC cells in a 24-h assay. With plocabulin, cells retained nearly 100% viability at 1 nM (concentration that corresponded to a complete inhibition of cell migration and invasion). Thus, the inhibitory concentrations of plocabulin on HUVEC migration and invasion were not coincident with those for inhibition of cell proliferation, indicating that the aforementioned effects were not likely mediated through unspecific cytotoxicity of the drug.

### Effects of plocabulin in HUVEC capillary tube structures in three-dimensional collagen matrices

**Fig. 4:**
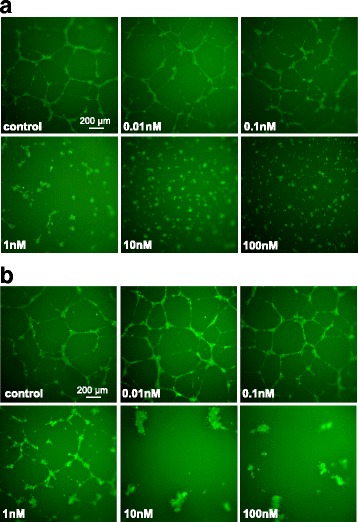
Antiangiogenic and vascular-disrupting effects of plocabulin. **a** Inhibition of de novo angio-tube formation; HUVEC cells were seeded on top of a Matrigel layer and incubated in the presence or absence of plocabulin for 24 h; cells were stained with calcein-AM and photographed by fluorescence microscopy. **b** Disruption of angio-tubes; HUVECs were seeded on top of a matrigel layer, as described, and treated with different concentrations of plocabulin; cultures were stained with Calcein-AM (green) and photographed after 24 h of incubation

To analyze how plocabulin affects the formation of new capillaries, HUVEC cells were grown on Matrigel in the presence or absence of the compound. Untreated, control HUVEC cells rapidly formed a network of angio-tube like structures that were visualized by fluorescence microscopy after staining the cells with calcein-AM (Fig. 4). Plocabulin interfered with the correct formation of the HUVEC capillary network at concentrations as low as 0.1 nM (Fig. 4a). At higher concentrations, the intercellular connections were mostly absent with drastic effects on the formation of the capillary network. Having established that plocabulin interferes with the formation of capillary networks from HUVEC cells when cultured on Matrigel, we wondered if the compound was also affecting an already established capillary network. At the concentration of 0.1 nM some effects on the capillary network could be observed (Fig. 4b). Treatment with plocabulin 1 nM or higher concentrations resulted in a significant disruption of the formed capillary-like network, with the majority of cells forming small clumps with a few number of intercellular connections. A complete dissapearance of the network was observed at concentrations higher than of 1 nM; in this case, cells appeared retracted and distinct cords were no longer observed. Altogether, these results indicate that plocabulin interfere with the formation and stability of the capillary networks formed by endothelial cells when cultured on a matrigel basement membrane matrix.

**Fig. 5:**
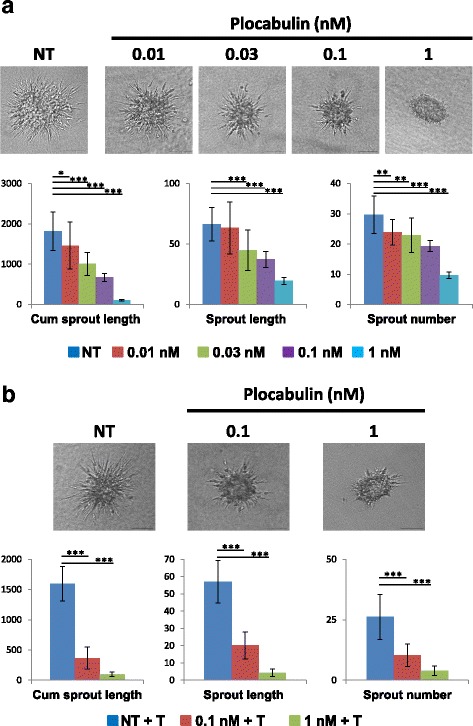
Plocabulin inhibits spheroid sprouting. **a** Representative images (upper panel) and quantification (lower panel) of spheroid sprouting assay as measured as cumulative sprout length (μm), sprout length (μm) and sprout number in the indicated conditions; data are shown as mean +/− standard deviation. **b** Representative images (upper panel) and quantification (lower panel) of spheroid sprouting assay in the presence of thymidine (T) as measured as cumulative sprout length (μm), sprout length (μm) and sprout number in the indicated conditions; data are shown as mean +/− standard deviation. Comparisons between different samples were analyzed by Student’s t test. Differences were considered significant at *P < 0.05, **P < 0.01 and ***P < 0.001 . Scale bars represent 100 μm

We finally performed a 3D sprouting assay in the presence of increasing concentrations of plocabulin. Twenty-four hours after embedding in collagen gel, non-treated spheroids of endothelial cells had the ability to produce and to extend capillary-like sprouts (Fig. 5). Addition of plocabulin affected the global angiogenic activity of endothelial cells in a concentration-dependent manner, as quantified by the mean cumulative sprout length per spheroid (Fig. 5a). This effect was the consequence of a reduced number as well as the reduced length of sprouts, indicating that plocabulin impeded both the formation of primary sprouts and their extension or stabilization (Fig. 5a). Treatment of pre-established angiogenic sprouts with the same drug concentrations lead to their collapse (data not shown). To determine if these effects were mediated by the antimitotic properties of the compound, we added thymidine in the medium to block cell division. Control spheroids treated with thymindine still actively produced long sprouts (Fig. 5b). Interestingly, treatment with plocabulin severely affected sprouting activities compared to the non-treated spheroids, precluding a mitosis effect of the compounds at the concentrations used.

### Plocabulin collapses tumor vessels in xenograft models

**Fig. 6:**
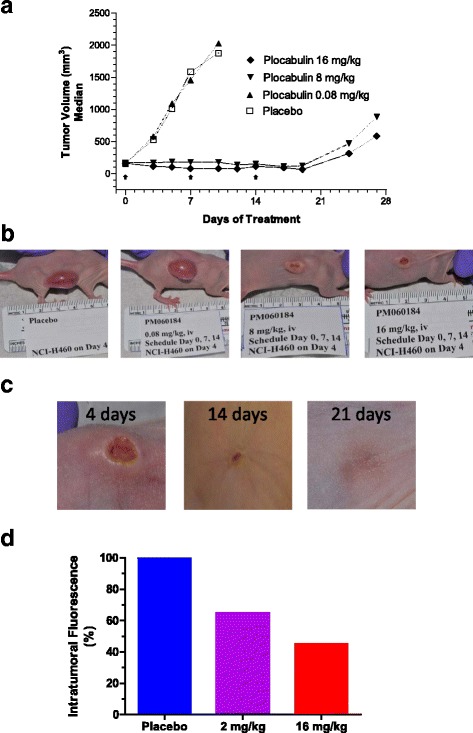
Antiangiogenic effects of plocabulin in tumor xenografts. **a** Tumor growth curve for mice bearing NCI-H460 lung xenografts and treated with placebo or plocabulin. Treatments were iniciated at a tumor volume size of ca. 150 mm^3^ and were intravenously administered at 0.08, 8 and 16 mg/kgfor three consecutive weeks (black arrows). Each point represents median values (*n* = 10). **b** Typical appearance of NCI-H460 lung xenografted tumors 4 days after the first administration of placebo or plocabulin (at 0.08, 8 and 16 mg/kg). **c** Evolution over the time of a tumor that achieved complete remission upon plocabulin treatment at 16 mg/kg .**d** Evaluation of vasculature in mice bearing NCI-H460 lung (*n* = 3/group) xenografts 24 h after the treatment with either placebo, 2 or 16 mg/kg of plocabulin (Angiosense™ 680 was dosed immediately after the treatment)

We then performed xenograft studies to test whether the in vitro antiangiogenic activity of PM060184 was also involved in its in vivo antitumor activity. NCI-H460 lung tumor cells were xenografted into the right flank of athymic nu/nu mice. Once the tumors reached ca. 150 mm^3^, the mice were randonmly assigned into groups of 10 mice each and either vehicle or plocabulin (0.08, 8 and 16 mg/kg/day) was intravenously administered for 3 consecutive weeks. At the drug doses used in the experiment, no significant toxicity or body weight loss was observed in the treated animals (data not shown). As shown in Fig. 6a, the highest doses of plocabulin presented antitumor activity with a statistically significant inhibition (*p* ≤ 0.0001 vs placebo) of tumor growth as well as, a rapid, extensive and irreversible hemorrhagic necrosis 4 days after the first dose (Fig. 6b). Complete tumor regressions were also observed after the administration of plocabulin at 16 mg/kg:Out of 10 mice, 1, 2, 3, 7 and 7 animals experienced complete tumor remissions on days 5, 7, 10, 14 and 21, respectively (Fig. 6c). We then studied if the observed effects on tumors were in part due to a reduction in functional vascular volume induced by the drug. For this purpose, randomly selected mice bearing H-460 xenografts were treated with a single dose of placebo, 2 or 16 mg/kg of plocabulin (*n* = 3/group). As shown in Fig. 6d, a strong and dose-dependent decreases in their vasculature as assessed by the percentage of reductionin the intratumoral fluorescence of plocabulin-treated animals (ca. 65 and 45% compared to placebo for 2 and 16 mg/kg, respectively) after the administration of Angiosense™ 680. Finally, the antiangiogenic activity of plocabulin was further evaluated in nude mice bearing MDA-MB-231 breast cancer xenografts (*n* = 6). Again, in this tumor xenograft, the administration of a single dose of plocabulin (16 mg/kg) induced a very strong reduction in the number of vessels after 24 h of treatment (Additional file 3: Figure S2). Altogether, these results confirmed the antiangiogenic activity of plocabulin.

## Discussion

It is well established that tumor growth and metastasis are angiogenesis-dependent and, hence, blocking this process has been considered as a strategy for arresting tumor growth [30]. This possibility has stimulated intensive research and the development of antiangiogenic molecules. Recent studies have demonstrated that MTAs present anti-angiogenic and/or vascular-disrupting properties [14, 16–20]. These observations have prompted the search for new MTAs with high selectivity for the tumor vasculature, and which would provide additional targets for cancer therapy. In this study, we show that plocabulin, a new microtubule depolymerazing agent, presents antiangiogenic and vascular-disrupting activities. By altering microtubule dynamics in endothelial cells, plocabulin not only inhibits the in vitro migration and invasion capabilities of endothelial HUVEC cells but also interferes with their abilities to induce the formation of 3D capillary-like networks as well as it disrupts pre-existed vessels. This rapid collapse of endothelial tubular networks in vitro occurs in a concentration-dependent manner and is observed at concentrations lower than that affecting cell survival. More important, the in vitro findings were confirmed in tumor xenografts.

Plocabulin belongs to a new family of tubulin-binding agents originally isolated from the marine sponge *Lithoplocamia lithistoides* [31]. This compound is currently produced by total synthesis and is under evaluation in clinical studies in patients with advanced cancer. We have previously reported that plocabulin is an inhibitor of tubulin polymerization with potent antitumor activity, including P-glycoprotein over-expressing tumors [26]. This outstanding activity is related to the ability of plocabulin to bind with high affinity to a new site in the β-tubulin plus end, thus inhibiting the addition of further tubulin subunits at sub-stochiometric concentrations [25, 27]. At higher concentrations, microtubules are also destabilized by the formation of assembly-incompetent tubulin-drug complexes with unassembled tubulin subunits. At any rate, plocabulin reduces microtubule dynamicity in tumor cells, affecting both interphase and mitosis [26]. In the first case, the compound induces a disorganisation and fragmentation of the microtubule network and the inhibition of tumor cell migration. In the second case, it induces the appearance of multipolar mitosis and lagging chromosomes at the metaphase plate. These effects correlate with prometaphase arrest and induction of caspase-dependent apoptosis or appearance of tumor cells in a multinucleated interphase-like state unrelated to classical apoptosis pathways.

We now show that plocabulin also presents antiangiogenic and vascular-disrupting activities. Interestingly, these effects were observed at concentrations that severely suppress microtubule dynamics but do not affect endothelial cell survival. The inhibition of microtubule dynamics induced by plocabulin is associated with subsequent alterations of total microtubule mass and changes in endothelial cell morphology. More interesting, it also affects the migration and invasion abilities of endothelial cells, both processes needed for a correct angiogenesis. Indeed, we observed that, in 3D in vitro models, plocabulin inhibited the sprouting of endothelial cells as well as tube formation. Alterations of the microtubule network in endothelial cells also affect and disrupt pre-existing angiogenic vessels. All these effects were confirmed in xenografted mice, and were evident within 24 h after treatment, and at doses below the MTD. The in vivo antivascular effects of plocabulin were characterised by a large reduction in vascular volume, producing vascular shutdown and induction of extensive necrosis in tumors. Image studies with a fluorescent probe that remains intravascular after administration also show extensive and irreversible vascular shutdown following a single dose of plocabulin and occurring in tumor tissue. These results are not surprising since, as detailed above, many crucial endothelial cell activities relevant to angiogenesis require a functional microtubule cytoskeleton [7, 8]. In addition, the morphological changes observed in plocabulin-treated endothelial cells could induce an increase of the vascular permeability, leading to high interstitial pressure and additional loss of blood flow. Moreover, the disruption of vascular network could result in the exposure of abnormal components of the basement membrane, which in turn can result in the induction of a coagulation cascade with subsequent thrombus formation and collapse of tumor vasculature. Altogether, these data suggested that an antivascular mechanism might, at least in part, contribute to the anti-tumor activities of plocabulin. These antiangiogenic effects could be achieved even at local concentrations lower than those necessary to cause a direct cytotoxic effect on tumor cells.

Other MTAs have been described as acting selectively on tumor blood vessels. Drugs such as taxanes, colchicines, combretastatins and vinca alkaloids were among the first chemotherapeutics reported to have anti-angiogenic or vascular-disrupting properties [13, 14, 17, 32–38]. These activities were related to their ability to affect microtubules on endothelial cells, altering cell adhesion, cell motility, and cell-cell interactions [34, 35, 39, 40]. For most of these agents, the effects on endothelial cells occur in vitro at low drug concentrations, which do not induce cell death, but affect microtubule dynamics implying that non-specific cytotoxicity does not play a role in the drug effect on vessel formation [13, 14, 16, 17, 33]. However, their therapeutic indexes appear to largely differ between them. In a first group of compounds, including colchicine or vinca alkaloids, the occurrence of vascular effects is observed at their MTD [29, 32, 41, 42]. A second group include MTAs in which the antiangiogenic effect is observed at doses lower than the MTD (e.g. vinflunine, combrestatin-A4, etc) [19, 21, 43]. Our results indicate that plocabulin should be included in this second group of MTAs. However, the chemical structure and biological properties of plocabulin differ significantly from combretastatin analogues or vinca alkaloids. The effects of plocabulin on tubulin dynamics in endothelial cells are also different from other MTAs [38, 44–47]. It was reported that vinflunine and paclitaxel increased microtubule dynamics in endothelial cells but not in tumor cells where they reduce it [36, 48]. In contrast, plocabulin reduced microtubule dynamics both in endothelial and tumor cells. Of note, we did not detect any changes on angiogenic-related proteins in supernates of endothelial or tumor cells after plocabulin treatment. This is an interesting effect as other anticancer agents (e.g. gemcitabine) increase the secretion of various growth factors, cytokines or prosurvival factors by endothelial or tumor cells, as a cell survival reaction that could induce the reversion of the antiangiogenic activity and vascular resistance [49, 50]

## Conclusions

We have demonstrated highly potent in vitro and in vivo antiangiogenic activities of plocabulin, and gained insight into its molecular mechanism of action. Our work here indicates that, additionally to direct effects on tumor cells, plocabulin induce a rapid collapse of newly formed capillary tubes and angiogenic vessels through altering microtubule dynamics that is required to maintain the shape of tubular networks and to execute major endothelial functions (e.g. migration and invasion). The effect does not appear to be a mere consequence of its antiproliferative activity, since all these effects are observed at concentrations that do not affect cell survival. We propose that the antiangiogenic property of plocabulin contributes to its antineoplastic activity.

## Additional files

Additional file 1: Figure S1. (A) Effects of plocabulin on HUVEC cell morphology and microtubule mass by fluorescence microscopy. Cells were cultured in the absence or presence of increasing concentrations of plocabulin at different time intervals. Cells were then stained for α-tubulin (red) and nuclei (blue). (B) Effects of plocabulin on HMEC-1 cell morphology and microtubule mass by fluorescence microscopy. Cells were cultured in the absence or presence of increasing concentrations of plocabulin at different time intervals. Cells were then stained for α-tubulin (red) and nuclei (blue). (PDF 340 kb)

Additional file 2: Table S1. Microtubule dynamics parameters for HUVEC cells treated with PM060184. EB3-GFP expressing HUVEC cells were exposed to 0.01, 0.03 or 0.1 nM plocabulin for one hour. Microtubule dynamics was then analyzed by confocal fluorescence microscopy. Kymographs of microtubule plus end dynamics were made and analyzed with the MTrackJ plugin running on the ImageJ software. Microtubule length changes ≥ 0.3 μm between two consecutive time points were considered as growth or shortening events, while changes < 0.3 μm were considered as pause events; only events starting and finishing within the recording were analyzed. Speed and distance were calculated for each growth event and were then averaged. Catastrophe frequency was calculated by dividing the number of catastrophes (transition from growth or pause to shortening) by the sum of growth and pause durations. For each condition, at least 10 microtubules per cell, in 10 cells in three independent experiments were analyzed. (DOCX 15 kb)

Additional file 3: Figure S2. Representative images and quantification of microvessel density in MDA-MB-231 breast tumor xenografts after a signle dose of plocabulin (16 mg/kg). Treatment started at a tumor volume size of ca. 500 mm^3^. Tumors were removed after 24 h and stained with hematoxylin/eosin. Data are shown as mean +/− standard deviation. Comparisons between different samples were analyzed by Student’s t test. Difference was considered significant at ***P < 0.001. (PDF 153 kb)

## Abbreviations

FBS: Fetal Bovine Serum
FGF: Human recombinant Fibroblast Growth Factor-basic
hEGF: human Epidermal Growth Factor
hFGF-β: human Fibroblast Growth Factor-Beta
HMEC-1: Human microvascular endothelial cells
HUVECs: Human Umbilical Vein Endothelial Cells
MTA: microtubule-targeting agents
MTD: maximum-tolerated dose
MTT: 3-(4,5-dimethylthiazol-2-yl)-2,5-diphenyltetrazolium bromide
PMA: Phorbol 12-myristate 13-acetate
R3-IGF-1: R3-Insulin-like Growth Factor-1
SRB: sulforhodamine B
VEGF: Vascular Endothelial Growth Factor
