# Supplementary figures and images for: Plocabulin, a novel tubulin-binding agent, inhibits angiogenesis by modulation of microtubule dynamics in endothelial cells
Source: BMC Cancer. 2018 Feb 7;18:164. doi: 10.1186/s12885-018-4086-2 (PMC5803861; doi:10.1186/s12885-018-4086-2)

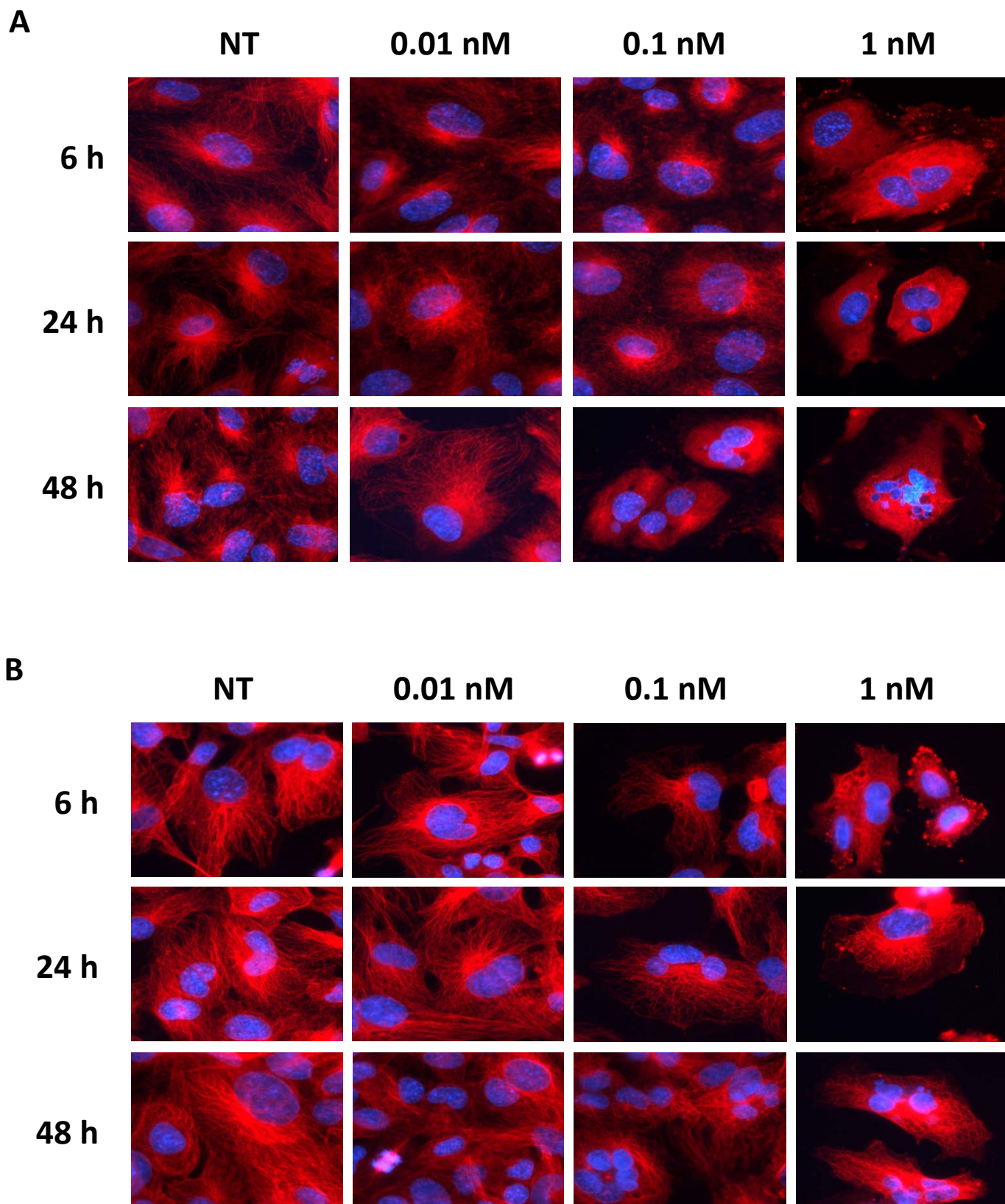

Figure S1

Supplement: Supplementary file 1 — (A) Effects of plocabulin on HUVEC cell morphology and microtubule mass by fluorescence microscopy. Cells were cultured in the absence or presence of increasing concentrations of plocabulin at different time intervals. Cells were then stained for α-tubulin (red) and nuclei (blue). (B) Effects of plocabulin on HMEC-1 cell morphology and microtubule mass by fluorescence microscopy. Cells were cultured in the absence or presence of increasing concentrations of plocabulin at different time intervals. Cells were then stained for α-tubulin (red) and nuclei (blue). (PDF 340 kb) [file 12885_2018_4086_MOESM1_ESM.pdf]

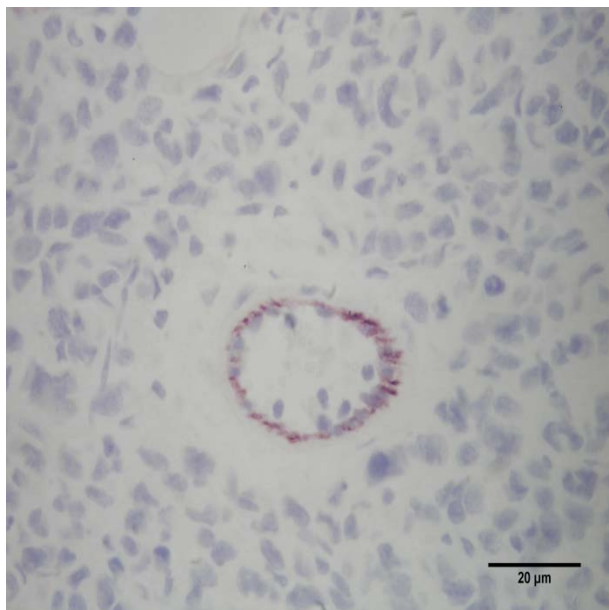

Placebo 100x

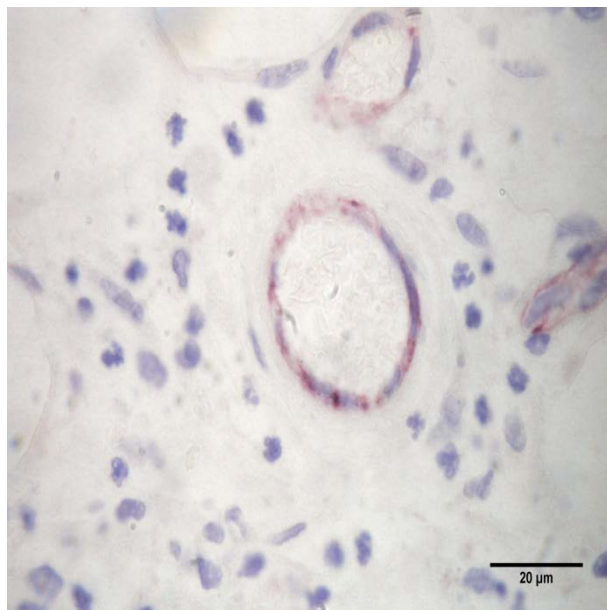

Plocabulin 100x

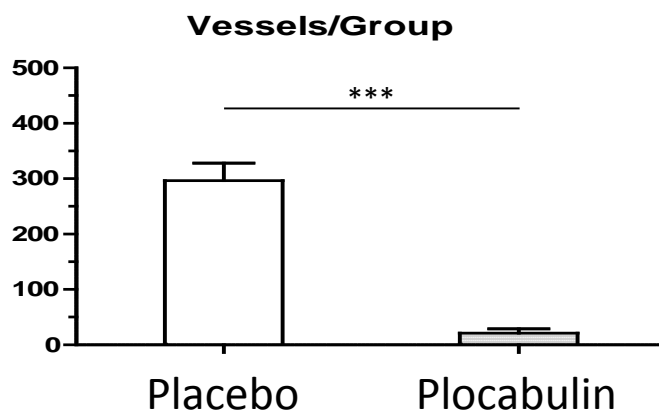

Figure S2

Supplement: Supplementary file 3 — Representative images and quantification of microvessel density in MDA-MB-231 breast tumor xenografts after a signle dose of plocabulin (16 mg/kg). Treatment started at a tumor volume size of ca. 500 mm3. Tumors were removed after 24 h and stained with hematoxylin/eosin. Data are shown as mean +/− standard deviation. Comparisons between different samples were analyzed by Student’s t test. Difference was considered significant at ***P < 0.001. (PDF 153 kb) [file 12885_2018_4086_MOESM3_ESM.pdf]
