# Supplementary material for: Plocabulin, a novel tubulin-binding agent, inhibits angiogenesis by modulation of microtubule dynamics in endothelial cells
Source: BMC Cancer. 2018 Feb 7;18:164. doi: 10.1186/s12885-018-4086-2 (PMC5803861; doi:10.1186/s12885-018-4086-2)
Supplement: Supplementary file 2 — Microtubule dynamics parameters for HUVEC cells treated with PM060184. EB3-GFP expressing HUVEC cells were exposed to 0.01, 0.03 or 0.1 nM plocabulin for one hour. Microtubule dynamics was then analyzed by confocal fluorescence microscopy. Kymographs of microtubule plus end dynamics were made and analyzed with the MTrackJ plugin running on the ImageJ software. Microtubule length changes ≥ 0.3 μm between two consecutive time points were considered as growth or shortening events, while changes < 0.3 μm were considered as pause events; only events starting and finishing within the recording were analyzed. Speed and distance were calculated for each growth event and were then averaged. Catastrophe frequency was calculated by dividing the number of catastrophes (transition from growth or pause to shortening) by the sum of growth and pause durations. For each condition, at least 10 microtubules per cell, in 10 cells in three independent experiments were analyzed. (DOCX 15 kb) [file 12885_2018_4086_MOESM2_ESM.docx]

# Table S1: Microtubule dynamics parameters for HUVEC cells treated with PM060184

|  | **Mean speed (µm/min)** | | **Mean distance (µm)** | | **Catastrophe frequency (min^-1^)** | |
| --- | --- | --- | --- | --- | --- | --- |
|  | Mean±SD* | % change | Mean±SD | % change | Mean±SD | % change |
| **NT**** | 18.5±3.2 | - | 2.2±0.2 | - | 1.9±0.3 | - |
| **0.01 nM** | 15.5±2.9 | -16.2 | 1.4±0.1 | -35.8 | 4.4±1 | +129.5 |
| **0.03 nM** | 11.9±3.5 | -35 | 1.1±0.2 | -47.2 | 5.2±0.7 | +172.6 |
| **0.1 nM** | 8.4±3.7 | -54.4 | 0.9±0.4 | -59.2 | 5.8±1.4 | +204.3 |

* SD: Standard Deviation, % of change: compared to the control

** NT: non-treated
